# Clean and fast cross-coupling of aryl halides in one-pot

**DOI:** 10.3762/bjoc.10.87

**Published:** 2014-04-22

**Authors:** Valerica Pandarus, Geneviève Gingras, François Béland, Rosaria Ciriminna, Mario Pagliaro

**Affiliations:** 1 SiliCycle Inc., 2500, Parc-Technologique Blvd, Quebec City, Quebec, Canada G1P 4S6; 2 Istituto per lo Studio dei Materiali Nanostrutturati, CNR, via U. La Malfa 153, 90146 Palermo, Italy

**Keywords:** borylation, Silia*Cat*, Suzuki–Miyaura, cross-coupling, one-pot

## Abstract

Unsymmetrically coupled biaryls are synthesized in high yield starting from different aryl bromides and bis(pinacolato)diboron by carrying out the Miyaura borylation reaction followed by the Suzuki–Miyaura reaction in the same reaction pot over 1–2 mol % Silia*Cat* DPP-Pd. The SiliaCat DPP-Pd catalyst is air-stable and the method does not require the use of inert conditions. The use of non-toxic isopropanol or 2-butanol as reaction solvent further adds to the environmental benefits of this new green synthetic methodology.

## Findings

Affording valued biaryls and heterobiaryls, namely ubiquitous chemical moieties in pharmaceuticals, natural products, photoactive species and many other functional molecules, the Suzuki–Miyaura cross-coupling reaction is widely employed by the fine chemicals and pharmaceutical industries [1]. The reaction involves a boron-containing nucleophile (a variety of aryl and heteroaryl boronic acids, esters, Ar-BBN, trifluoroborate and other boron species) and vinyl- or aryl halides as the electrophilic species. The use of the Suzuki–Miyaura reaction became routine both in industry and in research laboratories following Miyaura’s discovery from 1995. He demonstrated a direct route to boronic esters [2], namely the cross-coupling of bis(pinacolato)diboron (B_2_Pin_2_) with aryl or vinyl halides catalyzed by PdCl_2_(dppf) ([1,1′-bis(diphenylphosphino)ferrocene]palladium(II) dichloride) in the presence of excess KOAc at 80 °C in dioxane or in DMSO.

In contrast to a number of Pd catalysts that are air sensitive, PdCl_2_(dppf) is air stable. This made the method more versatile and provided a long awaited easy way to synthesize a broad range of boronic esters that are conveniently used in place of boronic acids in the Suzuki–Miyaura reaction when reactive functional groups are present in the electrophilic aryl halides [3–4].

Several homogeneous palladium catalysts of enhanced versatility for the cross-coupling of pinacolborane with aryl halides have since been introduced, for example by Buchwald and co-workers [5–6]. Of direct relevance to this research report, Giroux and co-workers reported the homogeneously palladium-catalyzed one-pot borylation/Suzuki coupling reactions already in 1997 [7].

Yet, it remains desirable to efficiently heterogenize palladium catalytic species used in cross-coupling reactions with the aim to prevent product contamination with palladium, reuse the expensive catalyst and streamline the whole synthetic process [8]. Furthermore, the replacement of DMSO or dioxane with a less toxic solvents is of obvious environmental and health relevance.

**Scheme 1:**
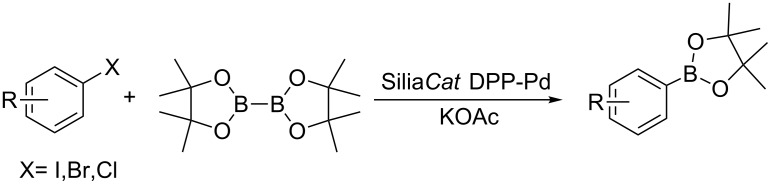
Heterogeneously catalyzed synthesis of boronic acid pinacol esters over Silia*Cat* DPP-Pd.

**Figure 1:**
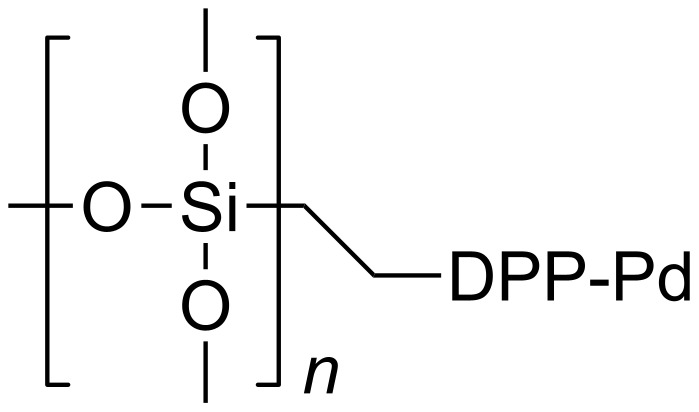
Chemical structure of Silia*Cat* DPP-Pd.

Sol–gel-entrapped catalysts are rapidly emerging as a promising tool for synthetic organic chemistry [9]. In this research context, we have recently described the heterogeneously catalyzed synthesis of boronic acid pinacol esters using a wide range of aryl chlorides, bromides and iodides and bis(pinacolato)diboron as borylating agent over the sol–gel-entrapped Silia*Cat* DPP-Pd catalyst (Scheme 1) [10]:

Silia*Cat* DPP-Pd is a commercially available catalyst [11] made of an organosilica matrix functionalized with diphenylphosphine ligands bound to Pd^2+^ (Figure 1).

The catalyst, which is highly active in C–C coupling reactions [12], has typical 0.2–0.3 mmol/g palladium loading, a high surface area and large accessible mesoporosity (300–650 m^2^/g, depending on the applied parameters of the sol–gel synthesis).

**Scheme 2:**
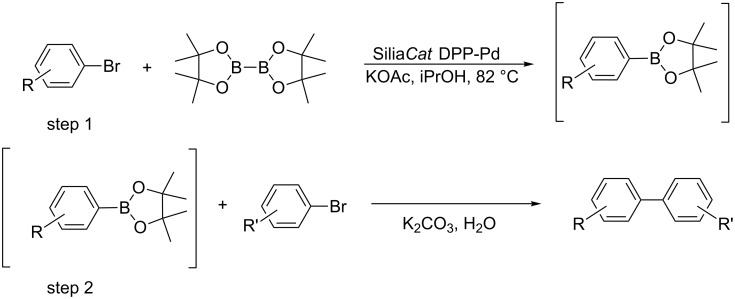
Heterogeneously catalyzed cross-coupling of two aryl bromides by consecutive borylation and Suzuki–Miyaura reactions, both of which are mediated by Silia*Cat* DPP-Pd.

The leach-proof and truly heterogeneous nature of Silia*Cat* DPP-Pd in the synthesis of boronic acid pinacol esters has been recently shown elsewhere [10], wherein the issue of catalyst recycling is also addressed in detail. Now we report a new method that establishes a direct route, in one-pot, to unsymmetrically coupled biaryls starting from two different aryl bromides and bis(pinacolato)diboron without the need to isolate the intermediate boronic acid pinacol ester. The overall process is described by Scheme 2.

The Silia*Cat* DPP-Pd catalyst mediates the borylation and the subsequent Suzuki–Miyaura reaction in an elegant one-pot sequential synthesis. Hence, an aryl bromide is first converted into the boronic acid pinacol ester (step 1 in Scheme 2). A different aryl bromide is then added along with aqueous base (step 2).

The reaction is carried out in iPrOH or in 2-BuOH. No work-up is performed after the borylation in the first step, nor is any catalyst added prior to conducting the second step of the sequence, the Suzuki–Miyaura reaction. Results in Table 1 show that, following aqueous work-up, different unsymmetrically coupled compounds are obtained in good to excellent yields by coupling numerous different aryl bromides with different aryl halides, including heteroatom-containing aryls.

**Table 1:** One-Pot Silia*Cat* DPP-Pd-catalysed borylation^a^ and Suzuki–Miyaura coupling^b^ reactions.

Entry	Substrate	Catalyst (mol %)	Base (equiv)	Solvent (M)^c^	Product	*t* (h)	Conv/select (Yield)^d^ (%)

1	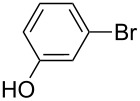	Silia*Cat* DPP-Pd 2	KOAc 2.2	iPrOH (0.75)	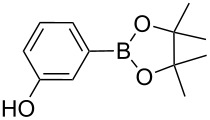	2	100/99
	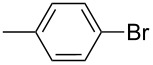	–	K_2_CO_3_ 2.3	+ 8 mL H_2_O	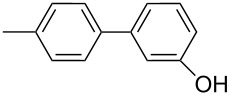	2 3	55 85

2	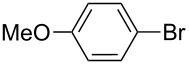	Silia*Cat* DPP-Pd 2	KOAc 2.2	iPrOH (0.75)	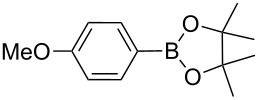	3	99/98
	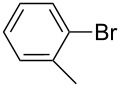	–	K_2_CO_3_ 2.3	+ 8 mL H_2_O	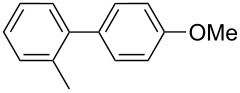	2 3	84.5 92/97 (88)

3	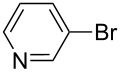	Silia*Cat* DPP-Pd 2	KOAc 2.2	iPrOH (0.75)	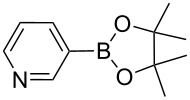	2 3	89 100/98
	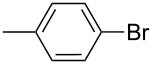	–	K_2_CO_3_ 2.3	+ 8 mL H_2_O	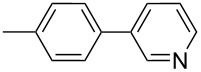	2 3	74 83

4	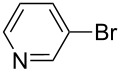	Silia*Cat* DPP-Pd 2	KOAc 2.2	iPrOH (0.75)	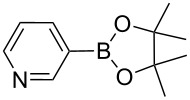	3	100/98
	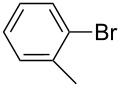	–	K_2_CO_3_ 2.3	+ 8 mL H_2_O	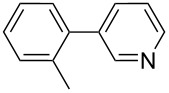	2 3	70 82

5	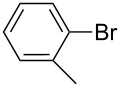	Silia*Cat* DPP-Pd 2	KOAc 2.2	iPrOH (0.75)	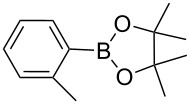	1 2	90 100/99
	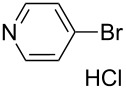	–	K_2_CO_3_ 2.3	+ 8 mL H_2_O	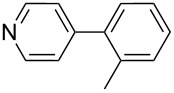	1 3	81 99/95 (89)

6^e^	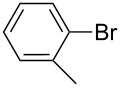	Silia*Cat* DPP-Pd 1	KOAc 2.2	2*-*BuOH (1.00)	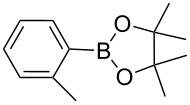	1 2	98 100/99
	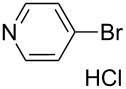	–	K_2_CO_3_ 2.3	+ 8 mL H_2_O	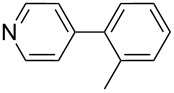	1 3	85 100/98 (94)

7^e^	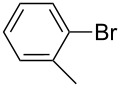	Silia*Cat* DPP-Pd 1	KOAc 2.2	2*-*BuOH (1.00)	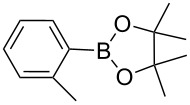	1 2	98 100/99
	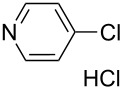	–	K_2_CO_3_ 2.3	+ 8 mL H_2_O	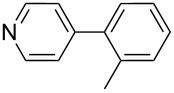	3 17	35 86

8^e^	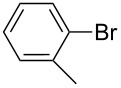	Silia*Cat* DPP-Pd 1	KOAc 2.2	2*-*BuOH (1.00)	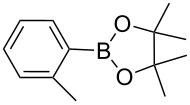	1 2	98 100/99
	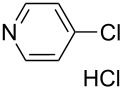	Silia*Cat* DPP-Pd 1	K_2_CO_3_ 2.3	+ 8 mL H_2_O	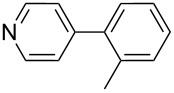	1 3	63 95

Experimental conditions. ^a^Step 1: Substrate 1 (10 mmol, 1 equiv), B_2_Pin_2_ (11 mmol, 1.1 equiv), KOAc (22 mmol, 2.2 equiv), 28 mL anhydrous iPrOH (0.75 M molar concentration with respect to reagents), 2 mol % Silia*Cat* DPP-Pd (0.25 mmol/g palladium loading), at 82 °C. ^b^Step 2: Substrate 2 (12 mmol, 1.2 equiv relative to substrate 1), K_2_CO_3_ (23 mmol, 2.3 equiv relative to substrate 1), 8 mL distillated H_2_O (iPrOH/H_2_O, 3.5:1, v/v). ^c^Molar concentration with respect to the substrate and to B_2_Pin_2_. ^d^Conversion/selectivity in cross-coupling product evaluated by GC–MS. Yield of the isolated product is given in parentheses. ^e^The borylation reaction is carried out at 98 °C in 21 mL anhydrous 2-BuOH (1.0 M molar concentration with respect to the reagents.

In detail, entries 1 and 2 in Table 1 show that 3-bromophenol and 4-bromoanisole are entirely converted into the boronic acid pinacol ester in 2–3 h over 2 mol % Silia*Cat* DPP-Pd. The same aryl halides are coupled with 4-bromotoluene or 2-bromotoluene to form the Suzuki–Miyaura coupling product in, respectively, 85% and 92% yield in 3 h.

Table 1, entry 3 shows that 3-bromopyridine is entirely converted in the boronic acid pinacol ester in 3 h over 2 mol % Silia*Cat* DPP-Pd. The resulting boronic ester is then smoothly coupled with 4-bromotoluene in 83% in 3 h, or with 2-bromotoluene in 82% in 3 h (Table 1, entry 4), over the same amount of catalyst in the same reaction pot.

Table 1, entry 5 shows the excellent applicability of Silia*Cat* DPP-Pd to the borylation of sterically ortho-substituted 2-bromotoluene, which is quantitatively converted into the corresponding arylboronate in 2 h over 2 mol % catalyst. The latter arylboronate is then quantitatively coupled with 4-bromopyridine hydrochloride in 3 h in the same reaction mixture. Table 1, entry 6 shows that the same quantitative conversions are obtained for the same substrates by replacing isopropanol with 2-BuOH. Only 1 mol % Silia*Cat* DPP-Pd is enough to promote full conversion of the original halides into the unsymmetrically coupled product.

Results displayed in Table 1, entry 7 show that when 4-bromopyridine hydrochloride is replaced by 4-chloropyridine hydrochloride, only a 35% yield of the Suzuki coupling product is obtained in 3 h over 1 mol % Pd catalyst. The yield grows to 86% by prolonging the reaction time to 17 h. However, it is enough to add another 1 mol % Pd catalyst amount to the reaction mixture, after the borylation is complete, to observe 95% conversion into the Suzuki coupling product in 3 h (Table 1, entry 8).

In conclusion, unsymmetrically coupled biaryls can be synthesized in high yield starting from different aryl bromides and bis(pinacolato)diboron by carrying out the Miyaura borylation reaction followed by the Suzuki–Miyaura reaction over 1–2 mol % catalytic amount of the sol–gel entrapped catalyst Silia*Cat* DPP-Pd in the same reaction pot. There is no need to isolate the intermediate boronic acid pinacol ester, while the air stable sol–gel entrapped palladium catalyst does not require the use of inert conditions.

Finally, the use of isopropanol or 2-butanol as reaction solvents further points out the environmental benefits of the method. As the fine chemicals and pharmaceutical industries are eventually adopting green chemistry synthetic methodologies [13], this method provides both industries with a clean route to valued compounds that are widely used in many industrial sectors.
